# Effects of Alkaline Solvent Type and pH on Solid Physical Properties of Carrageenan from *Eucheuma cottonii*

**DOI:** 10.3390/gels9050397

**Published:** 2023-05-10

**Authors:** Rival Ferdiansyah, Marline Abdassah, Achmad Zainuddin, Revika Rachmaniar, Anis Yohana Chaerunisaa

**Affiliations:** 1 Doctoral Program, Faculty of Pharmacy, Padjadjaran University, Jl. Raya Bandung-Sumedang KM 21, Jatinangor 45363, West Java, Indonesia; rival14002@mail.unpad.ac.id (R.F.);; 2 Department of Pharmaceutics, Sekolah Tinggi Farmasi Indonesia, Jl. Soekarno-Hatta No. 354, Bandung 40266, West Java, Indonesia; 3 Department of Pharmaceutics and Pharmaceutical Technology, Faculty of Pharmacy, Padjadjaran University, Jl. Raya Bandung-Sumedang KM 21, Jatinangor 45363, West Java, Indonesia; 4 Department of Chemistry, Faculty of Mathematics and Natural Sciences, Padjadjaran University, Jl. Raya Bandung-Sumedang KM 21, Jatinangor 45363, West Java, Indonesia; a.zainuddin@unpad.ac.id

**Keywords:** carrageenan, *Eucheuma cottonii*, physical properties, solid state

## Abstract

The effects of alkali type and pH on the physical properties of carrageenan have been extensively studied. However, their effects on certain characteristics of solid-state properties of carrageenan have not been identified. This research aimed to investigate the effect of alkaline solvent type and pH on the solid physical properties of carrageenan isolated from *Eucheuma cottonii*. Carrageenan was extracted from the algae using NaOH, KOH, and Ca(OH)_2_ at pHs of 9, 11, and 13. Based on the results of preliminary characterization, including yield, ash content, pH, sulphate content, viscosity, and gel strength, it was found that all samples followed Food and Agriculture Organization (FAO) specifications. The swelling capacity of carrageenan based on the type of alkali was KOH > NaOH > Ca(OH)_2_. The FTIR spectra of all samples were in agreement with that of standard carrageenan. The molecular weight (MW) of carrageenan using KOH as the alkali followed the order pH 13 > pH 9 > pH 11, while using NaOH, the order was pH 9 > pH 13 > pH 11, and while using Ca(OH)_2_, the order was pH 13 > pH 9 > pH 11. The results of the solid-state physical characterization of carrageenan with the highest MW in each type of alkali showed that the morphology of carrageenan using Ca(OH)_2_ has a cubic shape and is more crystal-like. The order of crystallinity of carrageenan using different types of alkali was Ca(OH)_2_ (14.44%) > NaOH (9.80%) > KOH (7.91%), while the order of density was Ca(OH)_2_ > KOH > NaOH. The order of solid fraction (SF) of the carrageenan was KOH > Ca(OH)_2_ > NaOH, while the tensile strength when using KOH was 1.17, when using NaOH it was 0.08, and while using Ca(OH)_2_, it was 0.05. The bonding index (BI) of carrageenan using KOH = 0.04, NaOH = 0.02, and Ca(OH)_2_ = 0.02. The brittle fracture index (BFI) of the carrageenan was KOH = 0.67, NaOH = 0.26, and Ca(OH)_2_ = 0.04. The order of carrageenan solubility in water was NaOH > KOH > Ca(OH)_2_. These data can be used as the basis for the development of carrageenan for excipients in solid dosage forms.

## 1. Introduction

Carrageenan is a group of linear polysaccharides composed of galactic molecules with galactose as the main unit and is found in the cell walls of algae species (*Rhodophyceae*), such as *Eucheuma*, *Chondrus* and *Gargantia* [1,2]. This substance is part of a hydrophilic linear sulphated galactan, which is often used in various fields, such as κ-carrageenan, ι-carrageenan, and λ-carrageenan [3,4]. The main differences in carrageenan type are based on the source of algae species, the number and position of the sulphate ester group, and the 3.6-AG content [4,5]. 

Carrageenan extraction using alkaline solvents is widely used because it can improve the mechanical properties of the resulting carrageenan [3]. The type, concentration, and pH of alkaline solutions significantly affect the physicochemical characteristics of carrageenan [6,7,8,9,10]. Alkaline solvents include NaOH, KOH, and Ca(OH)_2_ [11,12,13]. Several studies have revealed that Na^+^, K^+^, and Ca^+^ ions affect the rheological properties, gel formation microstructures, molecular weights, gel strength, and morphology of carrageenan kappa [6,14,15]. Carrageenan, with low molecular weight, produces tensile strength, gel strength, and resistance to dynamic mechanical and thermal analysis [16]. The physicochemical characteristics of carrageenan determine its function as an excipient in pharmaceutical preparations, e.g., as a gelling agent, suspending agent, emulsifier, tablet matrix, binder, and disintegrating tablets or granules [17,18,19,20,21,22]. Carrageenan has the characteristics of being able to form gels, increase viscosity, absorb water, and expand [23,24,25,26,27].

Many studies have been carried out on carrageenan extracted using alkaline solvents, such as NaOH, KOH, and Ca(OH)_2_, with characterization that refers to the Food and Agriculture Organization (FAO) standards and applications to pharmaceutical dosage forms [3,10,11,12,13,28,29,30,31,32,33]. However, there has been no research on the comparison of the effect of alkaline solution concentration based on pH on the physical characteristics and solid-state properties. These characteristics are fundamental in determining the potential use of carrageenan as an excipient for tablets or other solid preparations [34]. The carrageenan used in this study was extracted from *Eucheuma cottonii*. Carrageenan from this type of algae has the best functional properties, especially in gel formation, compared to other types of carrageenan [35,36,37]. This research aimed to study the effect of alkaline solvent types and pH variations on the solid physical characteristics of carrageenan.

## 2. Results and Discussion

The carrageenan produced in this study was an odourless white to yellowish-white powder. 

### 2.1. Physical Characteristics

#### 2.1.1. Yields, pH, and Ash Content 

Based on Table 1, there is a pattern in the carrageenan yield extracted using variations in pH and types of alkaline solvent. Higher pH values of the alkaline solution used in extraction resulted in higher yield values due to the ability of alkaline solutions to increase the penetration of solvents into algae tissue cells. This result is in agreement with a previous study that stated that the higher the concentration of the alkaline solution used, the higher the amount of carrageenan extracted [29]. Alkalis can also accelerate the transformation of α-1.3 and β-1.4-glycosidic to 3.6-anhydrogalactose [7].

Table 1 shows that the resulting carrageenan pH meets the value required by the FAO, which is below 10 [38]. The measured pH did not have a specific pattern related to the use of variations in solvents or the alkaline pH used. The pressing process influences the pH of carrageenan in removing the alkaline solution from the gel structure. Based on Table 1, a high alkaline solvent pH resulted in greater ash content. This can be explained by the phenomenon that alkaline solutions contain K^+^, Na^+^, and Ca^2+^, which are inorganic substances and were not lost during the heating process. The value of the total ash content met the specification standards of the FAO, namely less than 15% and not more than 40%, which is calculated for dry carrageenan [38].

#### 2.1.2. Sulphate Content, Gel Strength, Viscosity, and Molecular Weight of Carrageenan

Carrageenan sulphate content (Table 2) had no specific pattern. This is because, at the end of the carrageenan extraction process, the sulphation process used potassium sulphate of the same concentration in each treatment, causing the sulphate content in carrageenan to be homogeneous. The carrageenan sulphate content (Table 2) met the specifications of the carrageenan standards from the FAO, which are less than 15% and not more than 40% [38].

The research by Astuti et al. (2017) found that the viscosity of carrageenan was also influenced by sulphate levels and was directly proportional to the content of sulphate. A higher sulphate content resulted in a higher viscosity [29], which is due to the ability of the sulphate group in carrageenan to exert a repulsion force between negative charges along the polymer chain. As a result, the molecular chain stiffens so that the viscosity increases [22,39]. This finding supports the viscosity data in Table 2, where KOH pH 13, NaOH pH 9, and Ca(OH)_2_ pH 13 revealed the highest sulphate levels among other pHs of the same type of alkali. Molecular weight (MW) also plays a role in increasing viscosity. The MW of polymers shows the average chain length of the molecule. The high MW of carrageenan causes a homogeneous distribution of sulphate groups on the polymer chain, resulting in a more repulsive force and thus increased viscosity [40,41]. The gel strength of carrageenan cannot be separated from the influence of sulphate content and the type of ions contained in the alkaline solvent used. Table 2 shows that KOH generally had higher gel strength values than NaOH and Ca(OH)_2_. Theoretically, this is due to the ability of K^+^ cations, which are stronger than Na^+^ and Ca^2+^ cations in intermolecular binding. K^+^ from KOH can increase gel strength due to its ability to increase ionic strength in carrageenan polymer chains, such that the dissolved intermolecular forces are even more remarkable in forming helical aggregation [3]. The atomic weight of potassium is greater than that of sodium. The number of charges of monovalent cations with large atomic weights can form double helical gels, while cations with low atomic weights are less capable of forming helical gels. Ca^2+^ has a greater atomic weight than K^+^, but calcium has a divalent charge, which causes the carrageenan gel formed from Ca^2+^ to be brittle [3,42].

Based on Table 2, KOH pH 13 has the largest MW compared to NaOH and Ca(OH)_2_. In addition to being affected by species and alkaline pH, carrageenan MW is also affected by sulphate content. NaOH showed the largest MW in NaOH pH 9, and Ca(OH)_2_ showed the largest MW in extracting Ca(OH)_2_ pH 13. When viewed from the sulphate content, KOH pH 13, NaOH pH 9, and Ca(OH)_2_ pH 13 have the highest sulphate contents compared to the others.

#### 2.1.3. Swelling Capacity of Carrageenan Extracted Using Various Types of Alkaline Solvents and pH

The carrageenan swelling is due to the electrostatic repulsion of the sulphate group contained in its chain. The negative charge of the sulphate group on different chains induces electrostatic repulsion. This causes the distance between the chains to increase so that the tissue space becomes more extensive and more permeable to larger molecules. Thus, water can penetrate the tissue such that there is an increase in the volume and weight of the carrageenan structure [27].

**Figure 1:**
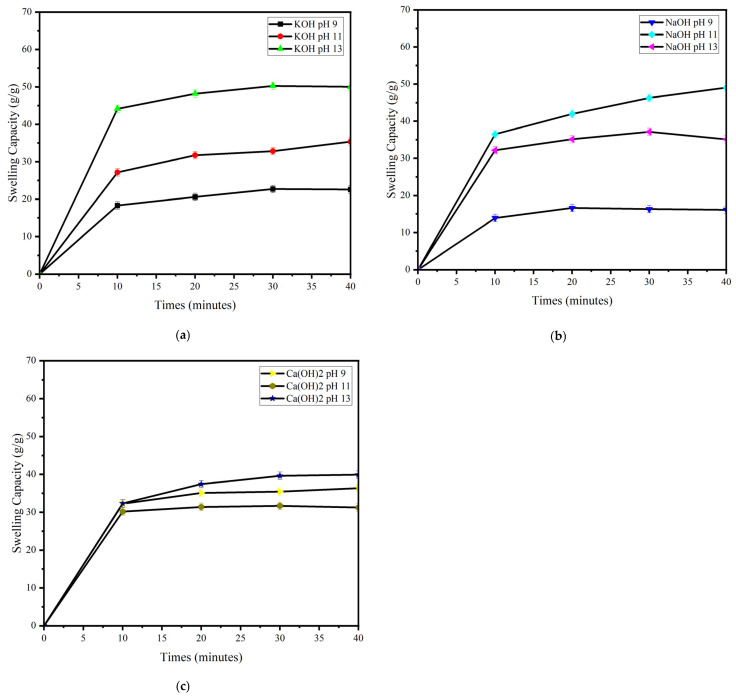
Swelling capacity of carrageenan: (**a**) KOH, (**b**) NaOH, and (**c**) Ca(OH)_2_, at pH 9, 11, and 13.

Figure 1 shows the difference in swelling capacity between carrageenan extracted using alkaline solvents at various pHs. Swelling capacity increases using KOH in the order pH 13 > pH 11 > pH 9, while for NaOH, the order was pH 11 > pH 13 > pH 9, and for Ca(OH)_2_, the order was pH 13 > pH 9 > pH 11. The difference in swelling levels is related to the sulphate levels, as listed in Table 2. 

Sulphate levels were shown successively as pH 13 > pH 11 > pH 9 when KOH was used, pH 13 > pH 9 > pH 11 for Ca(OH)_2_, and pH 9 > pH 11 > pH 13 when NaOH was used. Na^+^ is a monovalent cation, which is weaker than K^+^ and forms smaller helical bonds than KOH. In contrast, KOH has the most significant swelling ability compared to NaOH and Ca(OH)_2_ since K^+^ is an ion that can draw water into helical bonds so that the weight and volume of carrageenan become more remarkable than that of Na^+^ [21,43]. Meanwhile, the carrageenan extracted using Ca(OH)_2_ had the smallest swelling capacity because the Ca^2+^ ion formed a divalent bond with the sulphate group, resulting in a more compact and less flexible structure. As a result, this carrageenan tends to be less able to absorb water [39,44]. This is the reason for the slow increase in the swelling capacity of carrageenan extracted by Ca(OH)_2_. The type of cation and the concentration of sulphate groups in each carrageenan cause the difference in swelling levels, as can be seen in Figure 1 [21,39,43,44].

#### 2.1.4. FTIR Spectra of Carrageenan 

Carrageenan has a group of α(1.3)-D-galactose-4-sulphate and β(1.4)-3.6-anhydrous-D-galactose, sulphate group bond, and a cyclic carbon bond. Carrageenan contains sulphate groups, cyclic carbon, and an arrangement of α(1.3)-D-galactose-4-sulphate and β(1.4)-3.6-anhydro-D-galactose.

**Figure 2:**
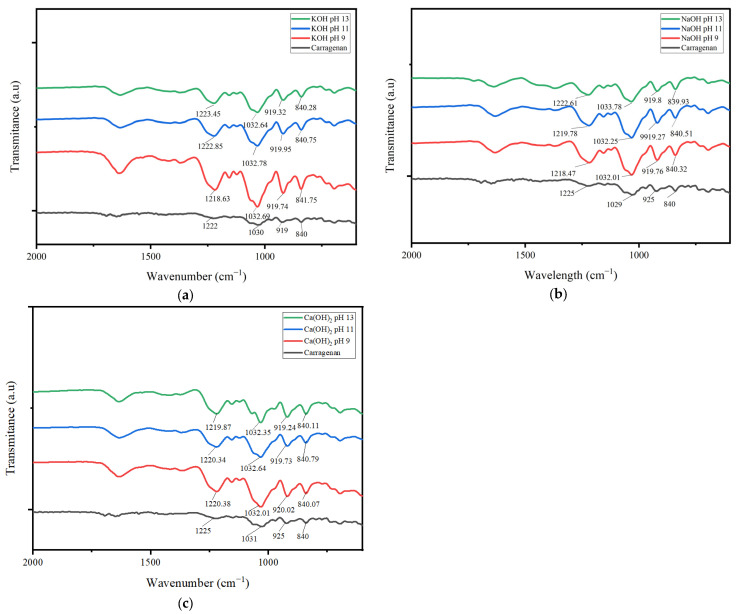
FTIR spectra of carrageenan extracted using various types of alkaline solvents: (**a**) KOH, (**b**) NaOH, and (**c**) Ca(OH)_2_ at various pHs (9, 11, and 13) compared to standard carrageenan.

Based on Figure 2, the carrageenan FTIR spectra showed a peak at wavenumbers 1220–1260 cm^−1^, i.e., the functional group S = O. The functional group S = O peaks were seen successively for KOH pH 9, 11 and 13, at 1218.63 cm^−1^, 1222.85 cm^−1^, and 1223.45 cm^−1^, for NaOH pH 9, 11 and 13 at 1218.47 cm^−1^, 1219.78 cm^−1^, and 1222.61 cm^−1^, and for Ca(OH)_2_ pH 9, 11, and 13 at 1220.38 cm^−1^, 1220.34 cm^−1^, and 1219.87 cm^−1^. 

The peaks of the spectrum showing cyclic carbon bonds were seen successively for KOH pH 9, 11, and 13, at wavenumbers 1032.69 cm^−1^, 1032.78 cm^−1^, and 1032.64 cm^−1^, for NaOH pH 9, 11, and 13 at 1032.01 cm^−1^, 1032.25 cm^−1^, and 1033.78 cm^−1^, and Ca(OH)_2_ pH 9, 11, and 13 at 1032.91 cm^−1^, 1032.64 cm^−1^, and 1032.35 cm^−1^. The 3.6-anhydrous-D-galactose carrageenan functional group was observed in the wavenumber range 915–928 cm^−1^. Peaks showing such functional groups were seen successively for KOH pH 9, 11, and 13 at 919.74 cm^−1^, 919.95 cm^−1^, and 919.32 cm^−1^, for NaOH pH 9, 11, and 13 at 919.76 cm^−1^, 919.27 cm^−1^, and 919.80 cm^−1^, and for Ca(OH)_2_ pH 9, 11, and 13 at 920.02 cm^−1^, 919.73 cm^−1^, and 919.24 cm^−1^.

The FTIR spectra for carrageenan at 835–850 cm^−1^ indicated the functional group D-galactose-4-sulphate. In Figure 2, it can be seen that for KOH pH 9, 11, and 13, the wavenumbers were successively 841.75 cm^−1^, 840.75 cm^−1^, and 840.28 cm^−1^, for NaOH pH 9, 11, and 13 at 840.32 cm^−1^, 840.51 cm^−1^, and 839.93 cm^−1^, and for Ca(OH)_2_ pH 9, 11, and 13 at 840.07 cm^−1^, 840.79 cm^−1^, and 840.11 cm^−1^. All carrageenan revealed FTIR spectra that corresponded to the carrageenan standard.

The most optimal carrageenan based on molecular weight and standard characteristics defined by the FAO were selected for further characterization, i.e., those from KOH pH 13, NaOH pH 9, and Ca(OH)_2_ pH 13. 

### 2.2. Evaluation of Solid Physical Characteristics

#### 2.2.1. Morphology 

**Figure 3:**
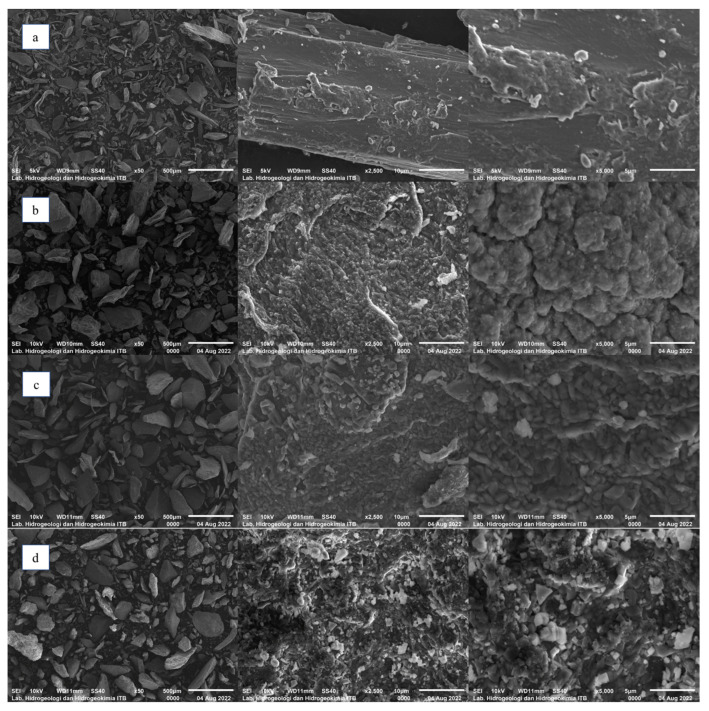
Morphological analysis of carrageenan: (**a**) Standard, (**b**) KOH pH 13, (**c**) NaOH pH 9, and (**d**) Ca(OH)_2_ pH 13 found using a scanning electron microscope.

Figure 3 shows the difference in surface shape between standard carrageenan and carrageenan extracted using various alkali types. The morphology of the standard carrageenan surface was smoother than that of extracted carrageenans. The surface morphology of KOH at pH 13 was spherical and irregular. The morphologies of NaOH pH 9 and Ca(OH)_2_ pH 13 tend to be crystal-like cubes. The role of cation types in carrageenan formation had a real impact due to the ability of cations to interact with sulphate groups in the carrageenan polymer chain and form various types of bonds depending on the type of cation [45]. K^+^ ions form strong helical bonds with carrageenan kappa compared to Na^+^ and Ca^2+^, thus affecting the morphology of carrageenan. Meanwhile, Na^+^ ions produce three different crystalline forms in several sulphate groups in various polymers. Ion Ca^2+^, a divalent cation, can change the helical bond more rigidly [44]. The use of different alkaline solvent types provides differences in the morphology of the carrageenan surfaces. The surface morphology of each carrageenan sample may indicate differences in physical properties, such as the percentage of amorphous and crystalline forms, density, and solid fraction.

#### 2.2.2. XRD Diffractogram of Carrageenan 

**Figure 4:**
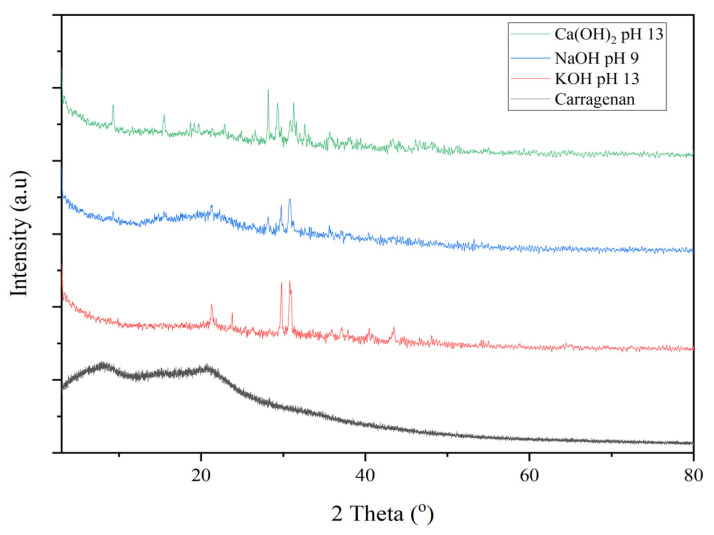
The diffractogram of carrageenan extracted using different alkaline solvents KOH pH 13, NaOH pH 9, and Ca(OH)_2_ pH 13 compared to standard carrageenan.

Figure 4 shows a diffractogram of standard carrageenan and the samples. The diffractograms of carrageenan samples using Ca(OH)_2_, NaOH, and KOH as alkaline solvents showed the presence of sharp peaks, which were not present in the diffractograms of the standard. In the results of the XRD chart analysis, produced using Match!3 software, the degree of crystallinity (DOC) of the produced carrageenan was highest for Ca(OH)_2_ pH 13 at 14.44% and its amorphous form at 85.56%, followed by NaOH pH 9, with a degree of crystallinity of 9.8% and an amorphous form of 90.16%, and KOH, with a degree of crystallinity of 7.91% and an amorphous form of 92.09%. Standard carrageenan does not indicate a peak in crystallinity and was predominantly in the amorphous form. The diffractogram supported the morphological observations shown in Figure 3, where the surface of carrageenan extracted by Ca(OH)_2_ pH 13 was a more crystal-shaped cube than that of NaOH, KOH, and of standard carrageenans. A relationship between monovalent and divalent cations is thought to play a role in forming helical bonds and other characteristics such as gel formation, rheology, depolymerization, swelling, and microstructure formation in the resulting carrageenan [43,44,45,46,47,48].

#### 2.2.3. Solid-State Physical Properties

- Density

Table 3 indicates that carrageenan extracted by Ca(OH)_2_ pH 13 had the most excellent density compared to that using KOH or NaOH. This can be explained by the higher molecular weight of Ca^2+^ in Ca(OH)_2_ compared to that of K^+^ and Na^+^.

- Solid Fraction (SF)

The solid fraction is the main factor that determines the binding strength of tablets that are from a material that is both brittle and elastic [49]. The value of the solid fraction can describe the compressibility of a material [50]. Generally, the SF value of material ranges between 0.6–0.8, with a target value ranging between 0.8–0.9 for commercial tablet manufacturing [51]. Small SF values represent the small proportion of the solid phase in a material. Conversely, the greater the solid fraction value, the greater the proportion of the deep solid phase. [52]. SF values outside that range indicate that the compressibility of the material is not good enough and is, therefore, not suitable for dominant use in a mixture, especially in a tablet or capsule dosage form. Based on Table 3, carrageenan extracted using KOH had the smallest value of solid fraction compared to that in Ca(OH)_2_ or NaOH. Ca(OH)_2_, as an alkaline solvent for carrageenan, gave the most considerable solid fraction and density values, which also revealed a more significant proportion of crystalline shape than other solvents used for carrageenan, as seen in Figure 4.

- Tensile strength (TS)

The tensile strength describes the tablet’s compactness of powdered material [49]. Materials with high tensile strength values make excellent binding materials [46,47,48]. Table 3 shows that carrageenan extracted using KOH has an enormous TS value compared to that of NaOH or Ca(OH)_2_. TS is inversely proportional to the solid fraction value of a material.

- Bonding index (BI)

The bond index is defined as the ability of a material to retain a fraction of the bond created during compression [49]. Generally, the bonding index ranges between 0.001–0.06 [40,49,53]. Table 3 indicates that carrageenan extracted using KOH had a BI value greater than that extracted using NaOH or Ca(OH)_2_. The value was influenced by the material’s morphological shape, surface area, and linearity with the value of TS.

- Brittle Fracture Index (BFI)

The brittle fracture index is used to measure the plasticity of a material [50]. Table 3 shows that carrageenan extracted using KOH had the largest BFI compared to that extracted using NaOH or Ca(OH)_2_. The value is directly proportional to the value of TS.

- Solubility

Based on Table 3, carrageenan extracted using NaOH had higher solubility than that extracted using KOH or Ca(OH)_2_. This phenomenon could be due to the carrageenan gelation containing Na^+^ ions being weaker than that containing K^+^ and Ca^2+^, resulting in a higher solubility in water [47].

## 3. Conclusions

The type of alkaline solvent and the pHs used in the extraction process affect the solid physical properties of carrageenan. The total ash content of all extracted carrageenans met the specification standards of the FAO (15–40%). Carrageenan extracted using KOH had the most significant swelling ability compared to that extracted using NaOH or Ca(OH)_2_. Carrageenan extracted using KOH pH 13, NaOH pH 9, and Ca(OH)_2_ pH 13 showed the highest MWs (289.80, 170.58, and 71.79 KDa, respectively) and the highest sulphate content compared to the others (20.64, 23.43 and 21.72%, respectively). These carrageenans were selected for further solid-state characterization. 

The surface morphology of each selected carrageenan indicated the differences in physical properties, such as the percentage of amorphous and crystalline forms, density, and solid fraction. Carrageenan extracted using Ca(OH)_2_ at pH 13 showed the highest degree of crystallinity (14.44%) compared to those extracted using NaOH pH 9 (9.8%) and KOH pH 13 (7.91%). The diffractogram supported the morphological observations where the surface of carrageenan extracted using Ca(OH)_2_ pH 13 was more of a crystalline-shaped cube than that extracted using NaOH pH 9 or KOH pH 13. Carrageenan extracted using Ca(OH)_2_ pH 13 had the highest density (2.02 g/mL) compared to that extracted using KOH pH 13 (1.94 g/mL) or NaOH pH 9 (1.94 g/mL). Carrageenan extracted using KOH pH 13 as the alkaline solvent had the smallest value solid fraction (0.42) compared to those extracted using Ca(OH)_2_ pH 13 (0.71) and NaOH pH 9 (0.70). KOH at pH 13 produced a carrageenan with a tensile strength of 1.17 MPa, using NaOH at pH 9, the TS was 0.08 MPa, and using Ca(OH)_2_ at pH 13, the TS was 0.05 MPa. Carrageenan using KOH pH 13 as an alkaline solvent had a bonding index and brittle fracture index value greater than that of NaOH pH 9 or Ca(OH)_2_ pH 13. The solubility of carrageenan using NaOH pH 9 was the highest (86.30%), which was greater than those using KOH pH 13 (68.60%) and Ca(OH)_2_ pH 13 (34.70%). These data can be used as the basis for the development of carrageenan as an excipient for solid dosage forms.

## 4. Materials and Methods

### 4.1. Materials

*Eucheuma cottonii* algae were obtained from Banggai Beach (South Sulawesi, Indonesia). Materials such as kappa-carrageenan (Tokyo Chemical Industry, Tokyo, Japan), sodium hydroxide (Merck, Jakarta, Indonesia), potassium hydroxide (Merck-Indonesia), calcium hydroxide (Merck, Jakarta, Indonesia), hydrogen peroxide (Merck, Jakarta, Indonesia), potassium chloride (Merck, Jakarta, Indonesia), potassium sulphate (Smartlab, Jawa Barat, Indonesia), barium chloride (Smartlab, Jawa Barat, Indonesia), acetic acid (Smartlab, Jawa Barat, Indonesia), hydrochloric acid (Smartlab, Jawa Barat, Indonesia), ethanol (Indoacidatama, Jakarta, Indonesia), and distilled water were used as received.

### 4.2. Methods

#### 4.2.1. Extraction

Fifty g of dried algae was soaked in 1500 mL of a mixture of 1% H_2_O_2_ solution and acetic acid pH 3.5 for 12 h, was then rinsed using running water, drained, and coarsely chopped. The algae were extracted using 1500 mL of alkaline solution at 80 °C and stirred at 150 rpm for 3 h. The alkaline solvents used in this study were NaOH, KOH, and Ca(OH)_2_ solutions with various pHs of 9, 11, and 13 [23]. Into 100 mL of extract, 7.5% K_2_SO_4_ solution was added and then stirred for 30 min. The extract was filtered when it was hot. The filtrate was cooled for 8 h at room temperature until the jelly form was obtained. The jelly was pressed using a hydraulic press to form sheets and then dried at 50 °C for 15–20 h. Dry carrageenan sheets were powdered and sieved using an 80-mesh sieve.

#### 4.2.2. Characterization of Carrageenan

The characterization was divided into two stages. The first stage was a general evaluation and characterization, referring to the FAO, including yield, pH, total ash content, sulphate content, gel strength, viscosity, molecular weight, swelling capacity, and functional group determination using FTIR.

The second stage involved further testing of the solid-state and physicochemical characteristics of three selected samples from each type of alkaline solvent, which were selected by their optimum results in the first evaluations. The second-stage tests included observing morphology, crystallinity, characteristics of the solid physical properties (density, solid fraction, tensile strength, bonding index, and brittle fracture index), and solubility.

#### 4.2.3. First Stage: General Characterization

- Yields

Yield measurements were conducted in accordance with the *Association of Official Analytical Chemists* (AOAC) procedures year 2005 [54].

- pH measurement

The pH measurements were conducted by following FAO procedures for 2014 [38], using a Mettler Toledo Model S220 SevenCompact pH meter.

- Total ash content

The ash content measurements were conducted by following procedures from the *Association of Official Analytical Chemists* (AOAC) [54].

- Sulphate content

The measurement of carrageenan sulphate levels was conducted by following Distantina et al.’s research procedures [55].

- Gel strength

Carrageenan gel strength testing was carried out by adopting a procedure performed in the Distantina et al. study [56].

- Viscosity

Viscosity testing was conducted by following Astuti et al.’s research procedures [29].

- Swelling capacity

The swelling ratio was determined using the method described by Rahmi et al. [54] with minor modifications. Carrageenan of known precise weight (Wo) was put into a nylon filter bag (Wn), immersed in a Petri dish containing aquadest, and every 10 min, the nylon bag was lifted and weighed (Wt). This test was carried out for 2 h at room temperature. The surface water on the carrageenan was wiped using a nylon filter and weighed. The swelling ratio (g/g) of carrageenan was then calculated using Equation (1) [57]:

(1) $$\mathrm{Swelling}\;\mathrm{ratio}\;\left(\frac{g}{g}\right)=\frac{\left(\mathrm{Wt}-\mathrm{Wo}-\mathrm{Wn}\right)}{\mathrm{Wo}}$$

- Molecular weight

Molecular weight measurements, were conducted by using the intrinsic viscosity measurement method [58].

- Study of functional groups with FTIR

The FTIR analysis was performed according to the method described by Al-Nahdi et al. [12] using an FTIR spectrometer Thermo Nicolet Is5 ID 3 ATR, equipped with a ZeSn ATR cell. 

#### 4.2.4. Solid-State Physical Characterizations

- Morphological observations

Carrageenan samples were analysed using a scanning electron microscope (SEM) JEOL 6510.

- Crystallinity

The crystallinity of carrageenan samples was analysed using an X-ray Difractogram PAN analytical X’Pert Pro PW3030/X0.

- Density

The density of the carrageenan samples was determined by using the fluid displacement method:

ρ = W/[(a + w) − b] SG(2)

where ρ = sample density in grams per cubic centimetre; W = sample weight in grams; SG = liquid paraffin specific gravity = 0.802; a = pycnometer + liquid paraffin weight in grams; and b = pycnometer + liquid paraffin + granule weight in grams [53].

- Solid Fraction (SF)

The solid fraction or the relative density of carrageenan samples was calculated from the compact carrageenan true density and apparent density. The apparent density was determined from the dimensions and weight of the sample and the true density [59].

(3) $$\rho r=\frac{\mathrm{Apparent}\;\mathrm{density}}{\mathrm{True}\;\mathrm{density}}$$

- Tensile strength (TS)

Breaking force (F) or crushing strength was the load measured at the point at which the tablet breaks under diametrical compression between two flat plates. Tensile strength is a fundamental measurement of the resistance to fracture. Breaking force can be converted into a tensile strength value,

(4) $$\mathrm{Tensile}\;\mathrm{strength}\;(\sigma )=\frac{2F}{\pi \mathrm{dh}}$$

where d is the diameter of the tablet, and h is the tablet thickness [50].

- Bonding index (BI).

The BI was determined by dividing the average tensile strength (σ) by the average hardness (P) [60]:

(5) $$\mathrm{Bonding}\;\mathrm{index}\;(\mathrm{BI})=\frac{\sigma }{P}$$

- Brittle fracture index (BFI)

BFI was measured by comparing the tensile strength (To) of a tablet with a centre hole with the tensile strength (T) of a similar tablet without a centre hole. The centre hole was a built-in model defect, which simulated the actual voids formed in the tablets (due to air entrapment) during manufacture. BFI was calculated using Equation (6) [61]:

BFI = 0.5 [(T/To) − 1](6)

- Solubility

The testing of the carrageenan sample solubility was performed by dissolving the sample by up to 1.5% using a beaker, referring to the method developed by Gontard and Guilbert with a slight modification. The sample was stirred with a magnetic stirrer for 15 min until uniformly mixed. The sample was then filtered with Whatman filter paper. The supernatant was removed, and the residue was dried at 105 °C for 24 h or 1 day [62].

(7) $$\mathrm{Solubility}=\frac{a-\left(c-b\right)}{a}\times 100$$

where a = sample weight (g), b = Whatman filter paper weight (g), and c = dry sample weight (g).

## Figures and Tables

**Table 1:** Yield, pH, and ash content of carrageenan extracted using various types of alkaline solvents and pH.

Alkaline Solvents	pH Solution	Yields (%)	Carrageenan pH	Total Ash Content (%)
KOH	9	25.69 ± 0.12	8.40 ± 0.03	26.82 ± 0.98
	11	27.72 ± 0.16	8.30 ± 0.05	33.17 ± 0.11
	13	30.65 ± 0.09	8.57 ± 0.08	36.41 ± 0.49
NaOH	9	23.37 ± 0.19	8.32 ± 0.06	22.51 ± 0.67
	11	29.73 ± 0.26	8.47 ± 0.02	24.37 ± 0.21
	13	34.51 ± 0.18	8.52 ± 0.06	36.28 ± 0.46
Ca(OH)_2_	9	26.62 ± 0.19	8.40 ± 0.03	33.05 ± 0.14
	11	28.56 ± 0.33	8.17 ± 0.04	36.34 ± 0.40
	13	32.87 ± 0.35	9.20 ± 0.10	37.64 ± 0.30

**Table 2:** Gel strength, viscosity, and molecular weight values of carrageenan extracted using variations in alkaline solvent type and pH.

Alkaline Solvents	pH Solution	Sulphate Content (%)	Viscosity (cP)	Gel Strength (g/cm^2^)	Molecular Weights (KDa)
KOH	9	18.24 ± 0.50	4.333 ± 94	137.93 ±13.44	165.07 ± 0.20
	11	19.11 ± 0.50	1.133 ± 94	53.34 ± 8.87	45.54 ± 0.00
	13	20.64 ± 0.30	5.933 ± 94	82.39 ± 5.97	289.80 ± 0.10
NaOH	9	23.43 ± 0.50	2.433 ± 47	44.92 ± 1.15	170.58 ± 0.30
	11	23.26 ± 1.10	1.167 ± 47	12.78 ± 0.02	37.29 ± 0.00
	13	21.63 ± 1.10	1.700 ± 82	52.37 ± 2.95	105.58 ± 0.70
Ca(OH)_2_	9	19.74 ± 1.60	350 ±41	38.51 ± 5.18	11.02 ± 0.00
	11	16.54 ± 1.10	1.067 ± 94	29.08 ± 2.39	7.59 ± 0.00
	13	21.72 ± 1.10	2.550 ± 41	56.26 ± 4.91	71.79 ± 0.30

**Table 3:** Solid-state physical properties and solubility of carrageenan.

Solid Physical Properties	KOH pH 13	NaOH pH 9	Ca(OH)_2_ pH 13
Density (g/mL)	1.94 ± 0.01	1.94 ± 0.03	2.02 ± 0.07
Solid Fraction	0.42 ± 0.03	0.70 ± 0.01	0.71± 0.01
Tensile Strength (MPa)	1.17 ± 0.02	0.08 ± 0.00	0.05 ± 0.00
Bond Index	0.04 ± 0.00	0.02 ± 0.00	0.02 ± 0.00
Brittle Fracture Index	0.67 ± 0.03	0.26 ± 0.01	0.04 ± 0.00
Solubility (%)	68.60 ± 1.80	86.30 ± 2.30	34.70 ± 2.90

## Data Availability

Not applicable.
